# Fixational eye movements and binocular vision

**DOI:** 10.3389/fnint.2014.00052

**Published:** 2014-07-07

**Authors:** Jorge Otero-Millan, Stephen L. Macknik, Susana Martinez-Conde

**Affiliations:** ^1^Department of Neurobiology, Barrow Neurological InstitutePhoenix, AZ, USA; ^2^Department of Neurology, Johns Hopkins UniversityBaltimore, MD, USA; ^3^Department of Neurosurgery, Barrow Neurological InstitutePhoenix, AZ, USA

**Keywords:** microsaccades, drift, disparity, fixation, ocular, amblyopia

## Abstract

During attempted visual fixation, small involuntary eye movements–called fixational eye movements–continuously change of our gaze’s position. Disagreement between the left and right eye positions during such motions can produce diplopia (double vision). Thus, the ability to properly coordinate the two eyes during gaze fixation is critical for stable perception. For the last 50 years, researchers have studied the binocular characteristics of fixational eye movements. Here we review classical and recent studies on the binocular coordination (i.e., degree of conjugacy) of each fixational eye movement type: microsaccades, drift and tremor, and its perceptual contribution to increasing or reducing binocular disparity. We also discuss how amblyopia and other visual pathologies affect the binocular coordination of fixational eye movements.

## Introduction

**Figure 1:**
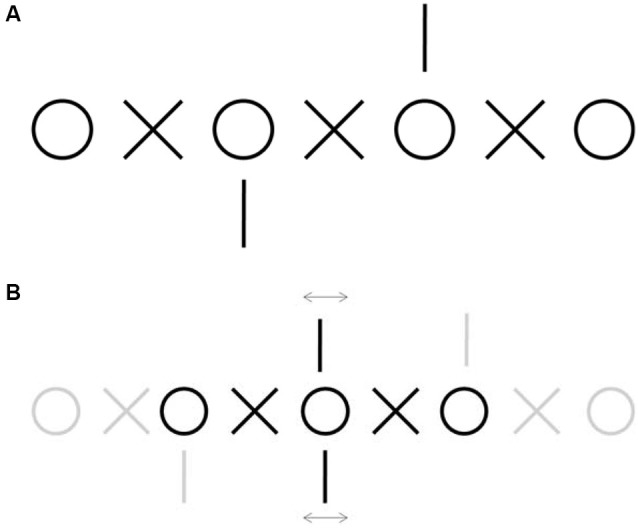
**Demonstration of fixation disparity. (A)** Example of nonius lines stimulus used to measure fixation disparity subjectively (Adapted from Jaschinski et al., 2005). Fixate on one of the central circles and diverge your gaze to achieve double vision, then try to match each circle with each circle and each X with each X. When you attain fusion, pay attention to the vertical lines. Misalignment between the top and the bottom line will be due to fixation disparity caused by fixational eye movements. **(B)** Schematic of the perception after fusion. Each of the central vertical lines is seen by one eye only and therefore do not fuse, whereas the central circles and Xs are seen binocularly. Fusion of the central circle indicates approximate alignment between the two eyes. Any simultaneous misalignment or motion of the vertical lines relative to each other will denote the corresponding fixation disparity.

Binocular vision is a sensorimotor process: eye movements work to keep the lines of sight of left and right eye pointing to the same target, and the visual system combines the resultant, slightly different retinal images, to form a single percept (i.e., binocular fusion) and create a sensation of depth (i.e., stereopsis). Correspondence between the left and right retinal images is complicated by the fact that our eyes are never perfectly still, even when we attempt to maintain our gaze on an object of interest. Small fixational eye movements change the degree of alignment between the two eyes and continuously move the retinal images (Figure 1). In spite of this constant motion, we rarely suffer from diplopia (double vision), indicating that the motor system and the visual system are finely tuned to each other. Thus, normal fixational eye movements do not preclude binocular fusion; in other words, fixation disparity (disagreement between the alignment of the left and right eye) stays below a certain threshold that would preclude fusion from taking place. In the presence of pathologies that interfere with proper functioning of the visual or motor mechanisms, such us amblyopia or strabismus, subjects may suffer from diplopia and lack stereoscopic vision. Here we review the oculomotor characteristics of binocular fixation, the perceptual consequences of fixational eye movements on binocular vision, and the clinical aspects of pathological instability during binocular fixation.

**Figure 2:**
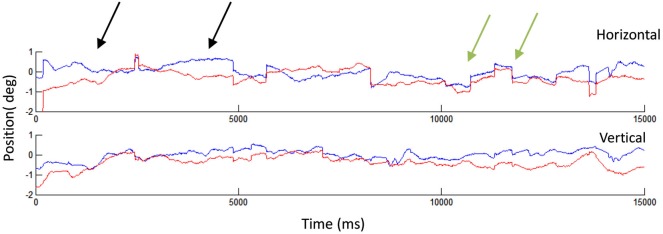
**Human fixational eye movements**. A 15 s recording showing microsaccades and drifts in the left (blue) and right (red) eyes. The black arrows indicate two instances of drift: in the first drift example (starting from the left), the two eyes start misaligned and finish aligned, whereas in the second example, the two eyes start aligned but finish misaligned. The green arrows indicate two microsaccades, which appear generally conjugate. That is, the two eyes move in the same direction and by a similar amount during each microsaccade. Eye movement recordings conducted with EyeLink 1000, SR Research.

The small eye movements that occur during attempted visual fixation consist of an alternation of quick motions called microsaccades (which occur once or twice per second) and periods of relative stability where the eye drifts slowly (Figure 2). A third type of fixational eye movement, beyond the measuring ability of most eye tracking systems, is called tremor, and is characterized by a very small quick oscillation that occurs simultaneously with drifts. Numerous studies have addressed the binocular properties of each kind of fixational eye movement. Most reports agree that microsaccades are generally conjugate, that is, that during microsaccades the two eyes move towards the same direction and by a similar amount, but there is less consensus about drifts.

From a perceptual standpoint, microsaccades have been shown to counteract visual fading and filling-in (Martinez-Conde et al., 2006; Troncoso et al., 2008a; McCamy et al., 2012; Costela et al., 2013), scan small and informative visual regions (Otero-Millan et al., 2008, 2013; McCamy et al., 2014b), improve visual acuity by precisely relocating the fovea (Ko et al., 2010; Poletti et al., 2013), and trigger perceptual transitions in a number of bistable illusions, including binocular rivalry (van Dam and van Ee, 2005; Troncoso et al., 2008b; Otero-Millan et al., 2012). Drifts and tremors are thought to enhance the processing of high spatial frequencies (Kuang et al., 2012).

## Binocular control of fixation eye movements

In this section we ask two main questions: First, are fixational eye movements conjugate? That is, do they have similar magnitudes and directions in both eyes? And second, does the difference in fixational eye movement directions and magnitudes between the two eyes serve to reduce or to increase fixation disparity? These are two related, but independent, questions. Disconjugate eye movements will reduce or increase disparity as a function of the vector difference between the movements in the two eyes, rather than of each absolute vector.

### Microsaccades

Microsaccades are small saccades that occur 1–3 times per second during attempted fixation. They tend to be less than 0.5° in amplitude, but can go up to 1° or more (Rolfs, 2009; Martinez-Conde et al., 2013; Otero-Millan et al., 2013).

Multiple studies, using different recording systems, have concluded that microsaccades are mostly conjugate eye movements (Krauskopf et al., 1960; Yarbus, 1967; St.Cyr and Fender, 1969; Schulz, 1984; Møller et al., 2002; Engbert and Kliegl, 2004). Indeed, most contemporary microsaccade studies use a binocular criterion (i.e., they only analyze microsaccades detected in both eyes) to reduce the amount of false positives resulting from the use of automatic microsaccade detection algorithms (Laubrock et al., 2005; Engbert and Mergenthaler, 2006; Engbert, 2006; Rolfs et al., 2006).

The first binocular recordings of microsaccades, performed in the early 1950s (Lord, 1951; Riggs and Ratliff, 1951; Ditchburn and Ginsborg, 1953), showed that a microsaccade in one eye was almost always accompanied by a microsaccade in the other eye, and that there was an overall correspondence between their respective magnitudes and directions.

Krauskopf et al. (1960) performed the first comprehensive and quantitative study of the binocular properties of microsaccades. They found that more than 95% of microsaccades had the same direction in both eyes and that the microsaccadic magnitudes in the two eyes were highly correlated. They showed that when the amplitude of the movement differed in the two eyes, the resulting difference tended to correct for errors in vergence. These results were later confirmed by St.Cyr and Fender (1969).

High-speed and high-resolution noninvasive video-trackers brought about a renewed interest in the binocular characteristics of fixational eye movements in the last decade. Møller et al. (2002, 2006) showed that microsaccades are generally conjugate. Engbert and Kliegl (2004) found that microsaccades tend to correct binocular disparity: on average, they reduced disparity by about 2 min of arc, with an approximate standard deviation of 6 min of arc. Around 35% of the microsaccades were error-producing, however. van Horn and Cullen (2012) recently showed that only 7–8% of monkey microsaccades have complete opposite directions.

Microsaccades and saccades are often immediately followed by a fast smaller movement in the opposite direction, called a dynamic overshoot. Dynamic overshoots are also saccadic in nature, i.e., they follow the same main peak velocity/magnitude relationship as saccades, and therefore differ from the glissades or vergence eye movements that can also occur after saccades (Kapoula et al., 1986). Dynamic overshoots can be monocular and tend to be more common in the abducting eye (Abadi et al., 2000). It remains unclear why overshoots are more common in the abducting eye, but it could be related to the fact that saccades are generally asymmetric, being slightly faster and shorter in the abducting eye (Collewijn et al., 1988). Due to an oscillation of the lens in the eye, dynamic overshoots may appear larger in recordings performed with videooculography or Dual Purkinje eye tracking systems than in recordings obtained with scleral search coils (Kimmel et al., 2012; Nyström et al., 2013).

### Drift

Drift refers to the slow eye movements that occur in between microsaccades during attempted fixation. Drifts are typically smaller and slower than microsaccades (typically less than 0.13 degrees in size, less than 0.5°/per second in speed (Rolfs, 2009)).

Eye drifts during fixation may not be a specific kind of eye movement, but result from the combined action of the gaze holding and retinal stabilization systems: The eye tends to drift slowly towards a “central position”, especially in the darkness and when fixating eccentric targets (Leigh and Zee, 2006). In the presence of a visual stimulus, the pursuit and optokinetic systems compensate for any retinal slip, and the vergence system compensates for binocular disparities. If the head is not completely fixed, the vestibulo-ocular reflex will moreover compensate for head movements. In addition, vergence eye movements or glissades can follow saccades (Kapoula et al., 1986). All these systems are subject to neural and sensory noise and thus may produce additional undesired drift.

Different studies have obtained discrepant results regarding the binocular coordination of drifts and its role in correcting fixation disparity: Ditchburn and Ginsborg (1953), reported that drifts are mainly conjugate in the vertical component, with the two eyes moving up or down simultaneously. The horizontal component presented lesser conjugacy, and sometimes had a “wave-like” appearance with alternating periods of convergence and divergence. Simon et al. found that drift appeared to occur synchronously between the two eyes, although sometimes diverging and sometimes converging (Simon et al., 1984). Multiple studies using different and complex analyses (Spauschus et al., 1999; Thiel et al., 2006, 2008) have found some level of synchronization between the two eyes during drifts. However, it is important to note that the fact that eye movements are synchronized according to these measurements is independent of the movements being conjugate and their effect on increasing or reducing disparity (Rolfs, 2009).

Research on the respective roles of drift and microsaccades on correcting fixation disparity has followed a similar path as studies on their role on correcting overall fixation position. Early on, Cornsweet et al. found that microsaccades, but not drift, had a corrective role in both overall fixation position and binocular disparity (Cornsweet, 1956; Krauskopf et al., 1960). Later studies found drifts to correct both fixation position (Steinman et al., 1967) and fixation disparity (St.Cyr and Fender, 1969), however. Specifically, St.Cyr and Fender (1969) found that drifts corrected errors in binocular disparity only in the horizontal direction. More recently, Engbert and Kliegl (2004) studied separately the contribution of microsaccades and drift to the correction of monocular fixation error and to the correction of binocular disparity, using random walk modeling and measuring the temporal correlations of eye positions for different timescales. They found that both microsaccades and drifts corrected fixation position on a long timescale (more than 100 ms), but only microsaccades corrected fixation disparity on a long timescale. Both microsaccades and drift produced random changes in disparity on short timescales (>20 ms).

### Tremor

Ocular microtremor is a small wave-like movement of just a few seconds of arc in amplitude and a frequency around 90 Hz (Martinez-Conde et al., 2004; Rolfs, 2009). Given tremor’s small amplitude and fast frequency, only the most accurate eye tracking systems are able to measure it (in most standard systems it falls within the noise level). Some specific devices have been developed to measure tremor (Bengi and Thomas, 1968; Bolger et al., 1992, 1999; McCamy et al., 2013a, 2014a). Early studies found that tremor was independent in the two eyes (Riggs and Ratliff, 1951), but more recent research has found a peak of energy in the spectral coherence of tremor in the two eyes, indicating some level of synchronization that could be due to motor neuron activity (Spauschus et al., 1999).

### Torsion

Human eyes have 3° of freedom: they can move not only horizontally and vertically, but also in the torsional plane. Torsional eye movements are rotations of the eye around the line of sight so the direction of gaze does not change. Torsional eye movements can induce disparities between the two eyes, especially in the periphery, and affect the 3D perception of slant (Enright, 1990). Van Rijn et al. measured spontaneous torsional eye movements during fixation and found that they were largely conjugate (Van Rijn et al., 1994). Cyclovergence, the difference between the torsional positions of the two eyes, was more stable than cycloversion, the average torsional position of the two eyes (0.07 vs. 0.2°). They also found that the presence of a background improved cyclovergence stability. Zhang and Li (2012) observed small torsional movements associated with microsaccades.

### Fixational eye movements in binocular vs. monocular viewing

Binocular performance can be superior to monocular performance of the same visual task, a phenomenon related to the brain’s ability to combine effectively the information from the two eyes, known as binocular summation. Binocular summation predicts improved fixation stability under binocular viewing as compared to monocular viewing. Accordingly, González et al. (2012) found increased fixation instability during monocular viewing, especially for the occluded eye. They also showed that microsaccade rate is lower during binocular viewing, in agreement with Krauskopf et al.’s previous finding that microsaccades are larger and less frequent during monocular viewing (Krauskopf et al., 1960). González et al.’s results are also consistent with the observation that subjects make larger and less frequent microsaccades when they fixate larger and less precise targets (Steinman, 1965; McCamy et al., 2013b). Motter and Poggio (1984) moreover found that binocular viewing of a fixation target in monkeys produced a small but consistent reduction in the variability of eye positioning, when compared to either eye alone. Other studies found that microsaccade properties did not differ for monocular and binocular viewing, however (Schulz, 1984; Nallour Raveendran, 2013).

### Fixational eye movements in near vs. far viewing

Few studies have performed direct measurements of the parameters of fixational eye movements at different viewing distances. One might expect such parameters to change with the vergence effort demanded at each distance. Krauskopf et al. (1960) found no differences in fixational eye movement characteristics between far and near viewing, however.

### Is disparity a stimulus for fixational eye movements?

The fact that microsaccades and drifts correct disparity on average does not necessarily mean that disparity information is used in microsaccade or drift generation. Disparity correction by fixational eye movements could be accomplished in two different ways. First, each eye could act independently to reduce its own fixation position error. Second, visual system’s estimation of disparity estimated could be used to produce a binocular eye movement that reduces such disparity.

Krauskopf et al. (1960) first set out to address this issue and found that both microsaccade magnitude and the probability of a microsaccade being triggered depended on gaze position error, but not on disparity error. Because Krauskopf did not find drifts to be corrective, he did not conduct similar analyses for drifts.

Later, St.Cyr and Fender (1969) confirmed Krauskopf’s microsaccade findings. They found that the microsaccadic correction of fixation position error did not improve when also considering disparity. However, they did find that disparity information contributed to drift control.

### Neural control of binocular fixational eye movements

Because microsaccades are very brief, they must be controlled without visual feedback (i.e., the time lag of visual feedback is longer than the duration of a regular microsaccade, i.e., <30 ms (Otero-Millan et al., 2008). Slow eye movements such as drift can be continuously controlled by visual feedback, however. These two types of control systems are commonly referred to as open-loop and closed-loop. Correspondingly, two different vergence systems, fast and slow, are said to control the binocular coordination of eye movements (Cullen and Van Horn, 2011). During fixation, these two systems might control the respective conjugacies of microsaccades and drifts.

Recent neurophysiological evidence indicates that microsaccades are generated by the same circuit as saccades (Hafed et al., 2009; Guerrasio et al., 2010; Hafed and Krauzlis, 2012; van Horn and Cullen, 2012). Neural control of saccade-vergence interactions has been controversial since the times of Hering and Helmholtz. Hering believed that both eyes were controlled by a combination of binocular commands of the same amplitude for each eye (Hering’s law of equal innervation (Hering, 1977)). In Hering’s framework, apparently monocular or disconjugate eye movements were explained by the mathematical combination of version and vergence movements. Helmholtz believed instead that the two eyes were controlled independently, and that binocular coordination was a learned behavior (Coubard, 2013).

**Figure 3:**
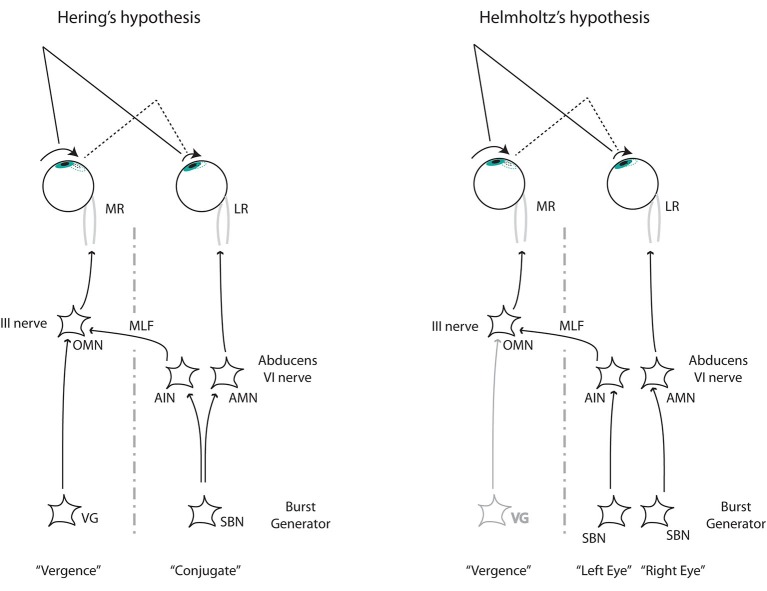
**The two alternative mechanisms to control disconjugate saccades**. Left: Hering’s hypothesis requires two independent commands (conjugate and disconjugate) that combine at the level of the oculomotor nucleus (OMN). Right: Helmholtz’s hypothesis requires separate monocular commands arriving at the abducens nucleus from the burst neurons at the paramedial pontine reticular formation (pprf). From Cullen and Van Horn (2011). LR, lateral rectus. MR, medial rectus; OMN, ocular motor neuron; AIN, abducens inter neuron; AMN, abducens motor neuron; VG, vergence; SBN, saccadic burst neuron.

Horizontal eye movements are controlled by motor neurons in the abducens nucleus (innervating the lateral rectus) and in the oculomotor nucleus (innervating the medial rectus). Neurons in the abducens nucleus project through the medial longitudinal fasciculus (MLF) onto the contralateral oculomotor nucleus. Thus, during a saccade towards the right, neurons in the right abducens and left oculomotor nucleus will show very similar discharge patterns driving the movements of the right and left eyes respectively. In this circuit, the controversy regarding Hering’s and Hemholhtz ideas translates into two possible implementations of disconjugate saccades. Following from Hering’s law, a third group of neurons should modulate the discharge of neurons in the oculomotor nucleus, whereas Helmholtz’s proposal requires two populations of neurons in the abducens nucleus, each corresponding to one eye (Figure 3). Zee et al. have proposed several models implementing both possibilities (Zee et al., 1992).

Studies have provided evidence in support of both Hering and Helmholtz’s proposals. Neurons in the mesencefalic reticular formation (MRF) encode vergence commands and project to the oculomotor nucleus (Mays, 1984). Other research has shown neurons in the abducens nucleus encoding the monocular saccadic command (Cullen and Van Horn, 2011), a finding that also applies to microsaccades (van Horn and Cullen, 2012). One possible explanation for these apparently contradictory results is that, whereas the slow vergence is controlled by the vergence neurons in the MRF, saccades are encoded monocularly in the abducens (Cullen and Van Horn, 2011; Coubard, 2013). A recent study has shown that neurons in the rostral superior colliculus, typically associated with conjugate eye movements only, also encode changes in vergence angle (Van Horn et al., 2013).

The generation mechanisms of tremor are unknown, but some studies have proposed that it originates in the ocular motor neurons (OMN; Spauschus et al., 1999). If so, the synchrony between the left and right eye tremor reported by some studies, may result from the synchrony among the motorneurons that drive each eye.

## Fixational eye movements and binocular perception

### Stereopsis and fixation instability

Our visual system creates the perception of depth based on the small horizontal differences between the images projected onto each eye. This phenomenon is called stereopsis and it requires that both eyes are directed to the same target, so that the two retinal images can be fused into a single percept. If the two eyes are not properly aligned, double vision (diplopia) occurs.

The fact that our eyes move continuously during fixation and we rarely suffer from diplopia limits the possible mechanisms responsible for stereoscopic vision. Thus, to study binocular fusion, stereopsis and diplopia, one must know how much the misalignment between the two eyes varies during fixation, and how much misalignment will result in diplopia.

**Figure 4:**
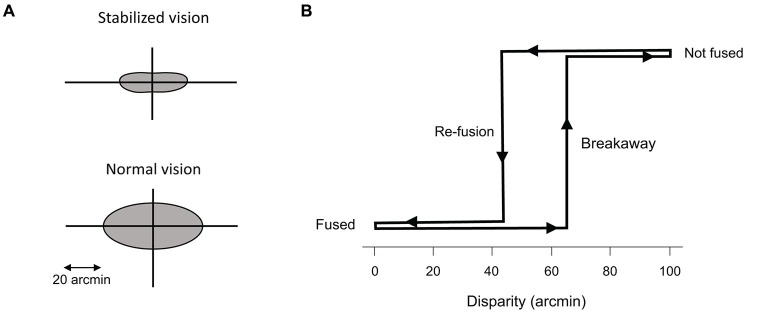
**Panum’s area. (A)** Representation of Panum’s area for stabilized and normal vision. **(B)** Hysteresis of fusion during retinal stabilization. The maximum disparity that allows fusion to start (re-fusion) is smaller than the amount of disparity that the system can maintain during fusion (breakaway), after fusion has already occurred. From Fender and Julesz (1967).

The maximum amount of disparity or misalignment between the two eyes that the visual system can fuse into a single percept is called Panum’s area, which is classically considered to range between 2 and 20 min of arc (Fender and Julesz, 1967; Duwaer and Brink, 1981a; Schor and Tyler, 1981). Panum’s area varies for different stimuli, and differs in the horizontal and vertical axis (Fender and Julesz, 1967; Qin et al., 2006). Fender and Julesz (1967), using retinal stabilization, found that the perception of stereopsis presents the properties of hysteresis (Figure 4). That is, once the visual system achieves fusion, the perception of stereopsis continues even if disparity increases, a phenomenon that is particularly noticeable with random dot stereograms. In such cases, the maximum disparity permitting fusion is only around 7 min of arch, but once fusion is achieved, the perception of stereopsis continues while separating slowly the two stimuli up to 2°. In normal viewing conditions (i.e., without retinal stabilization) the two values are much closer to each other, because the visual system will use vergence movements to correct the disparity. With respect to fixational eye movements, Fender and Julesz (1967) concluded that hysteresis could compensate for the disparity introduced by slow drift (but not for large disparities caused by fast microsaccades, which could only be corrected with vergence movements).

The standard deviation of disparity during human fixation is between 1 and 7 min of arc, depending on the study (Duwaer and van den Brink, 1981b; Steinman et al., 1982), which would mean that the sensory system is capable to achieve fusion with disparities between 3 and 21 min of arc (3 standard deviations) to avoid diplopia during normal vision. This values are comparable to Panum’s area’s measurements (2–21 min of arc) and consistent with variability across human studies (Duwaer and Brink, 1981a). In cases when eye movements introduce larger disparities, the hysteresis properties described above could help to maintain fusion for disparities of up to 2°. Motter and Poggio (1984) obtained larger values for the standard deviation of disparity during fixation in the primate (i.e., about 10 min of arc).

The continuous motion of the eyes and the related changes in disparity make it unlikely that the visual system relies solely on retinal correspondence between the left and the right eye to achieve stereopsis. At least two mechanisms have been proposed: One possibility, put forward by Anderson and Van Essen (1987) and supported by neuronal data from Motter and Poggio (1990), involves visual receptive field shifting based on a signal carrying eye velocity information (either from corollary discharge or from global motion estimation). Another mechanism, proposed by Howard and Rogers (1996), considers that the stereoscopic system only relies on first or higher order spatial derivatives of disparity. Small eye movements produce homogenous changes in disparity across the visual field, leaving the spatial derivatives unchanged. Because such a mechanism would be insensitive to small eye movements, it would not require additional signals to account for them and maintain fusion.

### Binocular rivalry

Binocular rivalry refers to the perceptual phenomenon that occurs when two very different visual stimuli are presented to each eye at corresponding retinal locations. In such cases, fusion does not take place, but the observer perceives an alternation of the two stimuli (rather than a mixture of both). Multiple studies have studied the potential relationship between eye movement production and the timing of the perceptual transitions in binocular rivalry (Sabrin and Kertesz, 1980; van Dam and van Ee, 2005, 2006a).

Binocular rivalry is present in stabilized images, arguing against a causal role of eye movements in driving the perceptual transitions (Blake et al., 1971; Wade, 1973) although the distribution of the durations of the intervals is different from the distribution during non-stabilized vision. Thus, it is unlikely that eye movements are the sole source of the transitions, but they may play a modulatory role. It is also possible that the transitions themselves affect the eye movements or that a third process of voluntary or involuntary control drives both the transitions and the eye movements.

Sabrin and Kertesz (1980) fount that microsaccade rates increased by 50% during binocular rivalry conditions vs. non-rivarly conditions, and that the increase happened mainly at the beginning of the periods of right eye dominance. Later, Sabrin and Kertesz (1983) found that simulated microsaccades with parameters matching real microsaccades while viewing stabilized rival stimuli best replicated the transitions occurring during non-stabilized viewing. This suggested that the oculomotor system and the rivalry system are tuned to each other. van Dam and van Ee (2005, 2006b) used orthogonal gratings as binocularly rivalrous stimuli, so that eye movements might produce retinal changes or not depending on their size relative to the grating frequency. They found that only microsaccades that led to retinal shifts were correlated with perceptual transitions during binocular rivalry.

## Fixational eye movements in amblyopia and strabismus

Ciuffreda et al. studied the fixational eye movements of subjects affected with amblyopia and strabismus. Their main finding was increased drift in the amblyopic eye during monocular viewing. If the amblyopia was due to strabismus, or in cases of alternating strabismus, the size and frequency of saccadic intrusions also increased (Ciuffreda et al., 1979a,b, 1980). More recently, Shi et al. (2012) found larger and less frequent microsaccades during monocular viewing with the amblyopic eye than during viewing with the fellow eye. In the case of viewing with the fellow eye, microsaccade parameters were comparable to those in subjects with normal vision. It is interesting to note that the characteristics of microsaccades during the fixation of large targets (i.e., in normal vision) (Steinman, 1965; McCamy et al., 2013b) resemble the microsaccadic parameters observed by Shi et al. in the amblyopic eye, suggesting that decreased fixation precision could be a common underlying mechanism.

**Figure 5:**
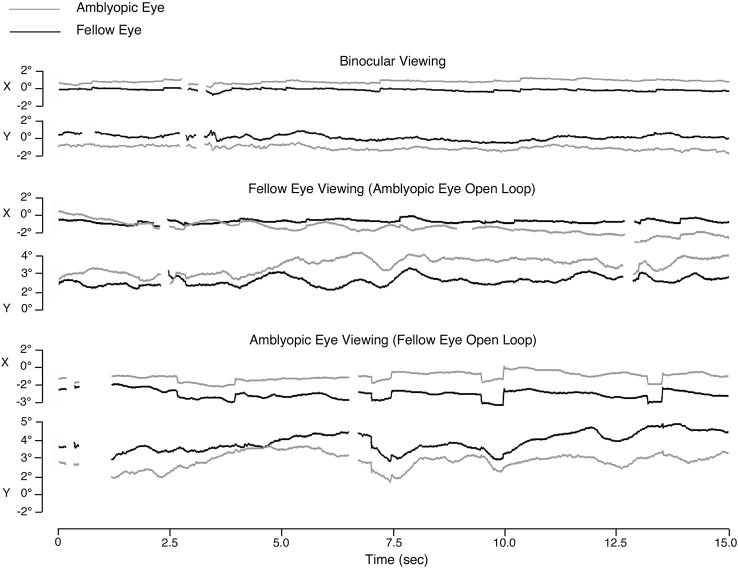
**Eye movements in a patient with strabismic amblyopia**. Horizontal position (X) and vertical position (Y) are plotted for the right eye in black and the left eye in gray. In this case the left eye is the amblyopic eye, and the right eye is the fellow eye (modified from González et al., 2012). Microsaccades become enlarged in both eyes during amblyopic eye viewing. Monocular viewing with the fellow eye results in increased instability in the amblyopic eye, whereas monocular viewing with the amblyopic eye results in increased instability in both eyes.

Fixation stability is another related eye movement metric affected by amblyopia. Fixation stability is typically measured as the dispersion of the eye position during attempted fixation, for example BCEA (bivariate contour elliptical area). This parameter combines the influences of microsaccades and drifts, however; thus it cannot differentiate between the effects of either eye movement. González et al. (2012) found decreased fixation stability in the amblyopic eye, when compared to the eyes of healthy observers. Fixation stability in the fellow eye (non-amblyopic eye) was comparable to that in healthy observers, under both binocular and monocular viewing. Monocular viewing with the amblyopic eye decreased fixation stability of the fellow eye as compared to the eyes of control subjects (Figure 5).

Amblyopia is typically accompanied by poor visual acuity. Subramanian et al. (2013) studied fixation instability in amblyopic eyes of children with strabismus and/or anisometropia (when the two eyes have unequal refractive power). They found that the BCEA was larger in the amblyopic eye than in the fellow eye, especially along the horizontal axis. Fixation instability was correlated with visual acuity, that is, patients with larger BCEA had lower acuity.

In an attempt to improve fixation stability for the amblyopic eye and achieve bifoveal fixation (Raveendran et al., 2014), reduced the contrast of the image shown to the fellow eye so that it was comparable to that perceived via the amblyopic eye. They found that, despite improvement in fixation stability in the amblyopic eye, bifoveal fixation is transient, with the strabismic eye drifting away from foveal alignment.

Another abnormal pattern of binocular fixational eye movements in amblyopic patients is fusion maldevelopment nystagmus syndrome (FMNS; Birch, 2013). FNMS is characterized by a horizontal conjugate nasalward slow-phase and a corrective temporalward quick-phase.

Recent research has shown that fixation instability can be used to detect amblyopia in early ages (when it can go undetected up to a third of the times (Loudon et al., 2011). This study used a binocularity score that measured how well subjects could fixate with both eyes on a target.

## General discussion

We have reviewed current knowledge about fixational eye movements in relation to binocular vision. An important recurrent theme is the two-way interaction between sensory systems and motor systems. Both sensory and motor aspects must be taken into account when studying visual perception and eye movement control: eye movements affect the sensory input, and the sensory input affects the eye movements in turn. The mutual tuning of fixation instability and the fusional system is a prime example of this interface. The relationship between fixation instability and decreased visual acuity in amblyopia is also indicative of the tight bond between the motor and the sensory facets of fixational eye movements.

Contemporary videooculography techniques are non-invasive and easy to operate, but do not have the same level of precision and accuracy as the scleral-search coil technique (McCamy et al., 2014a). To calculate the vergence position of the eyes one must determine the difference between the positions of the two eyes. Thus, vergence uncertainty will always double the uncertainty of the monocular eye position. This poses a challenge to the accurate measurement of disparity, and new studies conducted with videooculography techniques tend to report larger disparity values than those found in earlier research. Special care should be taken in calibrating the eye tracking set up in order to study vergence eye movements (De Luca et al., 2009).

Whereas recent studies have shed light on the generation and roles of microsaccades (see Martinez-Conde et al., 2013, for a review) much less is known about drift. Future studies should clarify the current discrepancies in results and determine how each of the gaze holding systems (vestibular, optokinetic, vergence or common integrator) contributes to drift. Can drift be generated purposely (i.e., to maintain a certain degree of disparity)? Is drift mainly a miss-calibration of either system (i.e., such as a lower gain in the integrator or an inappropriate gain of the optokinetic or vestibular systems)? Or is drift a mere manifestation of the noise level of one or all of those systems?

## Conflict of interest statement

The authors declare that the research was conducted in the absence of any commercial or financial relationships that could be construed as a potential conflict of interest.
